# Preparation and Properties of SiC-W_2_B_5_/C Composites

**DOI:** 10.3390/ma18092007

**Published:** 2025-04-29

**Authors:** Bo Xiao, Xia Zhang, Yongzhao Hou, Fagang Wang, Guangwu Wen

**Affiliations:** 1 School of Chemistry and Chemical Engineering, Shandong University of Technology, Zibo 255000, China; xiaobosdut587@sina.com (B.X.); 18409350572@stumail.sdut.edu.cn (X.Z.); 2 School of Materials Science and Engineering, Shandong University of Technology, Zibo 255000, China; houyz1990@sdut.edu.cn (Y.H.); 15615838760@sina.cn (F.W.)

**Keywords:** SiC-W_2_B_5_/C, carbon/ceramic composite, reaction hot-pressing, mechanical properties, graphitization

## Abstract

SiC-W_2_B_5_/C composites were prepared through a novel and simple processing route (reaction hot-pressing). The density of composites increased from 96.1% to 99.2% with the increase in SiC content from 5 to 30 vol%, leading to significant improvements in mechanical properties such as flexural strength, fracture toughness, and Vickers hardness, which for the highest W15S30 composite were measured at 292.3 MPa, 6.12 MPa·m^1/2^, and 3.32 GPa, respectively. The fracture morphology exhibited a mixed fracture mode of transgranular and intergranular, along with a pull-out phenomenon, indicating a well-combined interface. The sintering process involved Si powder acting as a liquid-phase sintering agent, reacting with B_4_C to increase the B concentration and promote the generation of W_2_B_5_, as well as reacting with the carbon phase to generate a transition phase of Si-B-C. The presence of this transition phase enhanced the interface bonding strength and catalyzed the graphitization process. As the SiC content increased, the promotion effect became more pronounced, with the degree of graphitization increasing from 59.2% to 66.2%.

## 1. Introduction

W_2_B_5_/C composites have a wide range of applications in engineering, including heating materials, electronic materials, high-temperature heat exchangers, and so on, due to their excellent properties such as high strength, high thermal stability, and good electrical conductivity [1]. In our previous research [2], W_2_B_5_/C composites exhibited exceptional mechanical properties, achieving a maximum flexural strength of 256.5 MPa and a fracture toughness of 3.85 MPa·m^1/2^, and were seen to develop an antioxidant film on their surface during high-temperature oxidation, preventing the composites from achieving oxidation.

The current sintering process for W_2_B_5_/C composites is suboptimal [3]. Specifically, the sintering activity is relatively low, which results in poor plasticity during the sintering process [4]. This limitation increases resistance during densification, making it challenging to achieve highly dense composites [5]. Optimal performance is observed when the W_2_B_5_ content is at 30 vol%; however, this concentration also introduces drawbacks, such as excessive density. For instance, the W30P composite with 30 vol% W_2_B_5_ content exhibits a density of 4.89 g/cm^3^. In applications such as mold fabrication for the hot-bent glass industry, a higher density necessitates greater power for mechanical transmission, which subsequently leads to increased energy consumption [6,7].

To enhance the performance of W_2_B_5_/C composites, the incorporation of a second phase is commonly employed to improve both sintering behavior and mechanical properties, thereby yielding W_2_B_5_/C composites with superior performance and high practicality [8,9]. To augment sintering activity, the second phase can be introduced to enhance the activation of grain boundaries and volume diffusion, leading to increased density in composites and further elevating their overall performance [10]. Following the dispersion strengthening theory of composite materials, the presence of second-phase ceramic particles with higher strength at crack sites leads to effects such as crack deflection, bifurcation, or pinning [11]. These effects enhance the matrix’s ability to resist fractures, thereby improving strength. The performance of composite material is influenced by factors such as the relative content of second-phase particles and the matrix, particle size ratio, interface characteristics, and the preparation process [12,13]. Second-phase particles are incorporated in a manner that ensures physical and chemical compatibility with the matrix [14]. Commonly utilized secondary reinforcement phases include metals, carbides, and boride ceramics [15]. For instance, Chen Xiaodong [16] investigated the use of metallic Zr as a secondary phase binder for WB composites, examining the effects of varying Zr content on the mechanical properties. Deng Yusha [17] investigated the influence of TiC on the sintering behavior of WB_2_ composites. The results indicated that the density and mechanical properties of WB_2_ composites containing TiC were superior to those of W_2_B_5_ ceramics. The Zr element can prioritize the B element within the W-B-C reaction system, and through the in situ reaction can produce ZrB_2_ while consuming the B content in the reaction system, significantly affecting the formation of WB. But when Si is added, the C reaction is prioritized in the reaction system, thereby not interacting with the B content of the W-B-C reaction system.

Among these secondary reinforcement materials, SiC is commonly utilized as a sintering aid for ceramic materials, which is particularly notable for its exceptional properties, including high mechanical strength, good resistance to oxidation, low thermal expansion coefficient, and strong compatibility with matrix carbon materials [18,19]. These characteristics make SiC a highly beneficial reinforcing material, widely utilized in demanding environments such as high temperature, high pressure, and corrosion in various industrial sectors [20]. The second phase typically serves two primary functions [21,22]: firstly, the ions from the second phase replace carbon, resulting in an electron and vacancy deficit, which augments the sintering driving force. Secondly, the second phase can function as a liquid phase, facilitating the movement of boron carbide particles, increasing their surface energy and thereby enhancing the sintering driving force.

The incorporation of SiC into W_2_B_5_/C composites is an effective strategy for enhancing their density and performance [23]. Additionally, Si is positioned adjacent to B and C, which suggests that based on the principles of similarity and compatibility [24,25], the presence of SiC will enhance the diffusion rate of B atoms and facilitate the reaction sintering of W_2_B_5_ [26]. During the sintering process, SiC not only promotes the densification of W_2_B_5_/C composites but also enhances their mechanical properties [27]. Consequently, SiC-W_2_B_5_/C ceramics are regarded as high-strength, high-temperature-resistant materials with significant application potential across various industrial sectors [28].

This study focuses on the development of SiC-reinforced W_2_B_5_/C composites. In comparison to the preparation methods of SiC, we used Si powder reactive sintering in a W_2_B_5_/C composite system, where Si powder was used as the starting material for synthesizing SiC through an in situ reaction and acted as a high-temperature liquid phase, facilitating the rearrangement of reactant phases, enhancing solid-phase dissolution and reprecipitation, ultimately improving the densification of composites. Additionally, it positively influenced key parameters such as grain size and grain shape of the W_2_B_5_ ceramic phase. This paper also aims to investigate the impact of SiC as a reinforcing phase on W_2_B_5_ and the carbon phase, as well as the effects on microstructure and properties of W_2_B_5_/C composites.

## 2. Experimental

The raw materials utilized in this study included commercially available WC, B_4_C, Si powder, and calcined petroleum coke powder. The B_4_C powder, characterized by an average particle size of 1.5 μm and a purity of 99.8%, was sourced from Zhuzhou Diamond Boron Carbide Co., Ltd., Zhuzhou, China. The WC powder, with an average particle size of 2.2 μm and a purity of 99.9% was obtained from Xintai Xindun Cemented Carbide Co., Ltd., Xintai, China. Si powder, exhibiting an average particle size of 3 μm and a purity of 99.8%, was purchased from Dongguan Xintie Metal Materials Co., Ltd., Dongguan, China Lastly, the calcined petroleum coke, with an average particle size of 20 μm and a purity of 97%, was acquired from Henan Lingshou Chemical Co., Ltd., Zhengzhou, China. In this research, the carbon source in the raw material system was in excess, and Si powder was used as the starting material for synthesizing SiC through an in situ reaction. Following this, hot-pressing techniques were utilized to produce SiC-reinforced W_2_B_5_/C composites.

The total reaction can be summarized as follows:

8WC + 5B_4_C → 4W_2_B_5_ + 13C (1)

Si + C → SiC(2)

Based on reactions (1) and (2), we calculated the raw material ratio, which can be seen in Table 1. Following the experimental design ratio, the raw material powder and graded zirconia balls were loaded into the mill tank (Changsha Miqi material equipment manufacturing Co., Ltd., Changsha, China). The ball mill tank was constructed from PTFE, which can endure prolonged grinding without contaminating the powder. The detailed grinding stage was as follows: First, we weighed 100 g of raw material powder and placed it in a 250 mL PTFE ball grinding tank. Then, we added graded zirconia balls (Zibo Qiming new material Co., Ltd., Zibo, China) ensuring that the weight ratio of the balls to powder was 2:1. We then introduced ethanol as a dispersant to facilitate the dispersion. The ball mill was then set to operate in a planetary ball mill at a speed of 120 r/min, with a duration of 24 h, followed by drying and sieving. The screened raw material powder was then transferred into a graphite mold (Zibo Baofeng carbon material Co., Ltd., Zibo, China) for hot-pressing. The furnace used in this experiment was ZRY-15 produced by Jinzhou Boda high-temperature material equipment manufacturing Co., Ltd., Jinzhou, China. The sintering process involved heating the material to 2000 °C, applying a pressure of 30 MPa, maintaining the temperature and pressure for 30 min, and finally cooling the furnace upon completion. The sintering process was conducted under vacuum conditions, free from the presence of oxygen. In detail, the steps were as follows, with the hot-pressure reaction sintering process being primarily divided into five stages: (1) The pre-pressing stage occurred at room temperature, during which the powder was pre-pressed to prevent any loss of material powder during the sintering process. The pressure was increased to the designated value, and after holding this pressure for 5 s, the pre-pressing stage concluded, transitioning into the vacuum stage. (2) The vacuuming stage also took place at room temperature, where the vacuum level was reduced from atmospheric pressure to 0.1 Pa. (3) The heating stage involved raising the temperature to 2000 °C at a predetermined heating rate. (4) In the heat preservation and pressure maintenance stage, pressurization commenced once the temperature reached the specified level, followed by a 30 min period of heat and pressure maintenance. (5) Finally, in the cooling stage, pressure relief began after the heat preservation and pressure maintenance were completed. Once the pressure was relieved, the heating current was cut off, and the system relied on the circulating water in the furnace walls to facilitate cooling.

The Archimedean drainage method was used to determine the density of composites by measuring the weight of the material in air and while floating in water. The sintered sample was removed from the mold, and the surface was ground using a grinder (Huzhou Shuanglin grinding Co., Ltd., Huzhou, China). Before testing, the sample was washed with water, placed in an ultrasonic cleaner (Shenzhen Emik Technology Co., Ltd., Shenzhen, China), and subsequently cleaned with alcohol(Shanghai Aladdin Biochemical Technology Co., Ltd., Shanghai, China) for 5 to 10 min; then, it was dried in a vacuum drying oven (Shanghai Yiheng Equipment Co., Ltd., Shanghai, China) at 100 °C until a constant weight was achieved and, after, it was cooled to room temperature. An electronic balance (MS-TS) (Dongguan Shengxing equipment Co., Ltd., Dongguan, China) was employed to measure the mass of the sample in air, denoted as *m*_1_, and the mass submerged in distilled water, denoted as *m*_2_. The following calculation formula for density was applied [29]:

(3) $$\rho =\frac{m_{1}\rho_{1}}{m_{1}-m_{2}}$$

where *ρ* (g/cm^3^) is the actual density, *m*_1_ (g) is the weight of the dried sample in air, *m*_2_ (g) is the weight of the sample in water, and *ρ*_1_ (g/cm^3^) is the density of water.

The relative density of a composite material is determined by the ratio of its density to the theoretical density, and the calculation formula is as follows [30]:

(4) $$d=\frac{\rho_{2}}{\rho^{\prime }}\times 100\%$$

where *ρ*′ (g/cm^3^) is the theoretical density, *ρ*_2_ (g/cm^3^) is the actual density, and *d* (%) is the relative density of a composite.

Flexural strength was assessed using the three-point bending method on an Instron-1186 universal testing machine, with specimens measuring 3 × 4 × 40 mm^3^, with a 30 mm span and a loading rate of 0.5 mm/min [31]. Fracture toughness was evaluated using the single-sided notched beam method on the same machine, with samples of 2 × 4 × 20 mm^3^ with a 16 mm span, a notch width of 0.25 mm, a depth of 2 mm, and a loading rate of 0.05 mm/min [32], and 10 samples were measured in each group. The Vickers hardness was determined using an HV 50 Vickers hardness tester (Shanghai Qingbo Equipment Co., Ltd., Shanghai, China) by applying a load to the polished test surface. Phase analysis of the composite materials was conducted using an MSXD-3 X-ray diffractometer (Beijing Peking University Smart Microstructure Analysis and Testing Center Co., Ltd., Beijing, China). The X-ray source utilized a copper target Kα, with a loading voltage of 30 kV, a current of 30 mA, and a scanning speed of 2°/min [33].

A Hitachi S-4700 scanning electron microscope(Hitachi, Tokyo, Japan) was utilized for the examination of the morphology, particle size, microstructure, and fracture morphology of composite materials. Sample preparation involved sandpaper polishing (Huzhou Shuanglin grinding Co., Ltd., Huzhou, China) with various mesh sizes, grinding, polishing, cleaning with absolute ethanol, and gold spraying on the test surface [34]. The structure and interface composition of the composites were analyzed using a JEM-2100 transmission electron microscope (JEOL, Tokyo, Japan). An oxidation experiment was conducted on the material in a high-temperature furnace, with the sample size set at 10 × 10 × 10 mm^3^ [35]. The surface was polished and cleaned before gradually increasing the temperature from room temperature to a specified temperature at a constant heating rate. Once stabilized, the test sample was placed in a constant temperature area and its weight was recorded at specific intervals. Following the completion of the test, a phase composition analysis of the sample surface was performed using an X-ray diffractometer, while micromorphology was observed with a scanning electron microscope.

## 3. Results and Discussion

### 3.1. Reaction Products

**Figure 1:**
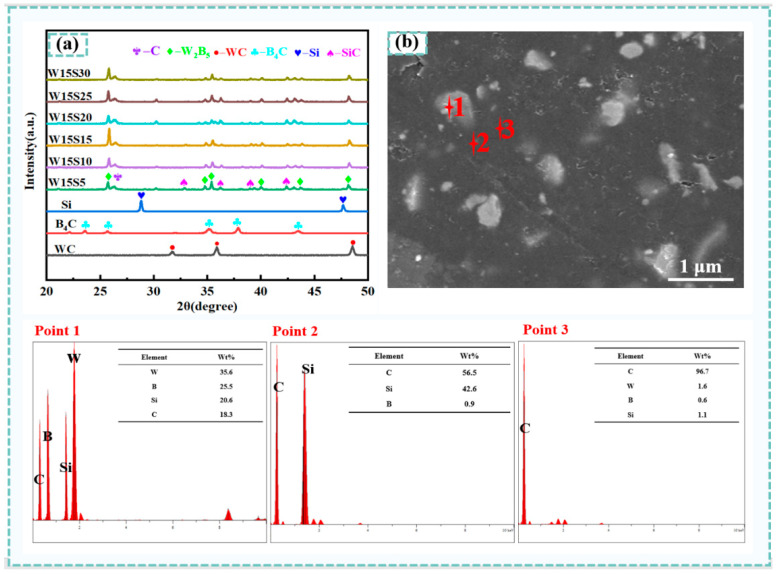
XRD patterns and EDS element analysis of SiC-W_2_B_5_/C composites. (**a**) XRD patterns of SiC-W_2_B_5_/C composites; (**b**) EDS element analysis of the W15S15 composite.

The physical phase composition of composites was identified, as depicted in Figure 1. XRD analysis of SiC-W_2_B_5_/C composites indicated that the composites were formed of different materials, with SiC additions ranging from 5% to 30%. An analysis of Figure 1a showed diffraction peaks corresponding to W_2_B_5_, SiC, and C in composites. Notably, the diffraction peaks of Si were not present, and clear peaks were observed, indicating the presence of SiC crystals [36]. The reaction between Si powder and C to form SiC was evident during sintering, as seen in Figure 1b, with a higher proportion of Si powder leading to increased SiC production.

Figure 1b presents the EDS analysis W15S15 composite. By integrating the XRD and EDS composition results, it was evident that the white particles corresponded to the W_2_B_5_ phase, the dark gray particles represented SiC, and the black regions indicated the C phase. The morphology of the W15S15 composite revealed that the C phase served as the matrix phase. Following the sintering process, a matrix skeleton was established. The W_2_B_5_ and SiC particles, which were generated during the reactive sintering process, functioned as reinforcing phases and were uniformly distributed within the matrix, effectively filling the voids between the C particles. Consequently, the W_2_B_5_ and SiC phases occupied the pores within the matrix, significantly decreasing the porosity of composites and facilitating its densification.

The reaction of Si in W_2_B_5_/C composites involved three pathways for the generation of SiC. The primary pathway was a direct reaction between Si and the added carbon source, resulting in SiC production. Additionally, Si can react with a portion of B_4_C or with the C produced from the reaction of WC and B_4_C. The following reactions are present in the reaction system [37].

(5) $$\left[Si\right]+\left[C\right]\to SiC$$

(6) $$\left[Si\right]+2B_{4}C\to SiC+\left[B\right]+\left[C\right]$$

(7) $$5B_{4}C+8WC\to 4W_{2}B_{5}+13C$$

The presence of liquid components in composite systems during sintering, attributed to the lower melting point of Si compared to sintering temperature, resulted in a combination of solid solution diffusion and dissolution–precipitation mechanisms. When sintering temperature exceeded melting point, the Si powder transitioned into a liquid phase, surrounding the WC and B_4_C particles to increase their contact area and facilitate the sintering reaction of W_2_B_5_. The liquid phase accelerated atom diffusion rates and reaction speeds. Moreover, as in reactions (5), (6), and (7), liquid Si weakened the covalent bond of B_4_C, leading to a reaction with C atoms to form SiC, while also precipitating B atoms and raising their concentration in the system [38]. The exothermic reaction between C and liquid Si triggered the sintering reaction process and aided in the formation of W_2_B_5_. The driving force behind the sintering process was surface tension and interfacial tension of liquid Si, which can be roughly classified into distinct stages [39].

The movement and rearrangement of solid particles during densification were influenced by the surface tension of liquid Si. Capillary forces formed in the wetting pores between solid B_4_C and WC particles, in addition to their own viscous flow, to accelerate this process. Liquid Si wetted the solid particles, filling the gaps and gradually eliminating the solid/gas interface in the reaction system. Pressure differences in pores of varying sizes drive the flow of liquid-phase Si between them. The penetration of liquid Si into the gaps created a component force that brought solid particles closer together due to tension. The curvature of liquid Si on concave surfaces of particles, influenced by differences in size and surface shape, resulted in unequal capillary and component forces acting on particle surfaces [40]. This accelerated the movement and rearrangement of particles in liquid-phase Si, ultimately promoting the formation of W_2_B_5_.

**Figure 2:**
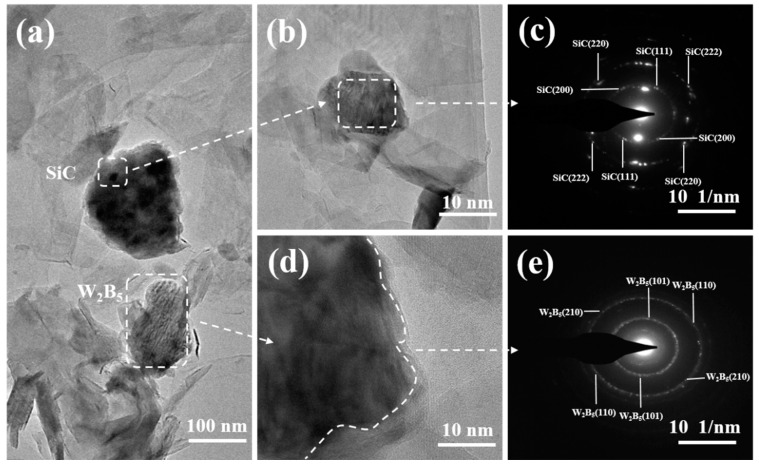
TEM micrographs of the W15S15 composites. (**a**,**b**) TEM of W15S15, (**c**) SAED images of SiC. (**d**) HRTEM images of W15S15, (**e**) SAED images of W_2_B_5_.

Solid-phase dissolution and reprecipitation processes occurred during the transformation. The B atoms located on the surface of solid B_4_C particles dissolved in liquid Si, resulting in the formation of a Si-B-C solid solution phase [41]. Due to higher saturated solubility and surface activity of B_4_C particles, they tended to dissolve more readily in regions with larger curvatures, leading to a smooth, flat, and roughly spherical particle surface. As the dissolution continued, some supersaturated atoms in the liquid Si phase precipitated on the surface of larger B_4_C particles. This dissolution and re-precipitation mechanism were facilitated by the migration of liquid Si within composites. A gradual transformation of reactant particles into a spherical shape occurred, along with a reduction in or disappearance of smaller particles and the growth of larger ones. Moreover, matrix particles adjusted their shape to enable closer packing, which enhanced the densification of composites. The TEM microstructure and electron diffraction patterns depicted in Figure 2 illustrated each phase, showcasing the layered structure of SiC and the well-integrated interface between W_2_B_5_ and SiC. The interface morphology indicated the generation of SiC within W_2_B_5_ grains as a result of a reaction between B_4_C and Si during sintering. Figure 2 presents HRTEM and SAED images of the W15S15 composite. Specifically, Figure 2a,b depict the black W_2_B_5_ ceramic phase and dark SiC phase, alongside the light gray C phase. Notably, the C phase formed a continuous matrix network structure, while the SiC and W_2_B_5_ phases were observed to wrap around the C phase interface, acting as enhanced phases. From Figure 2a, it is evident that the ceramic phase predominantly exhibited two morphologies: granular and plate-like. The crystal surface shown in Figure 2c corresponded to the diffraction pattern surfaces of SiC in the [111], [222], [200], and [220] orientations. Figure 2e illustrated the diffraction patterns of W_2_B_5_ in the [101], [111], and [210] orientations [42]. Figure 2d provided an HRTEM image, highlighting the interface between the W_2_B_5_ grains and the C grains. The interface between the ceramic phase and the carbon phase demonstrated good quality, indicating that the ordering of the carbon phases was satisfactory, which suggested that the crystallization of the carbon phase was complete and that graphitization was high. These findings were consistent with previous XRD analysis results. A well-defined interface was beneficial for enhancing the properties of composites.

The addition of Si powder impacted the graphitization of the carbon phase in composites. This experiment focused on SiC-W_2_B_5_/C composites with a high carbon phase content. In these composites, the carbon phase served as a continuous matrix, while the W_2_B_5_ and SiC in the ceramic phase were strategically dispersed throughout the carbon phase to enhance the overall performance of composites. A minor portion of the carbon phase was produced through the reaction of WC and B_4_C, whereas the majority was derived from calcined petroleum coke. XRD patterns show strong graphite (002) crystal plane diffraction peaks at 2θ = 26.5° for all composites. The sharp shape of the (002) crystal plane peak, along with the presence of (100) and (101) diffraction peaks, as well as (004) and (110) diffraction peaks, indicated a high degree of graphitization [43]. When Si powder melted, it entered the B_4_C unit cell, replacing C in the C-B-C three-atom chain within the B_4_C crystal structure to form a B-rich and Si-rich solid solution phase. The replaced C reacted with Si to form SiC, leading to continuous dissolution and release of C. The solid dissolution of Si into B_4_C resulted in a significant increase in lattice constant C. This dissolution caused the lamellar stacking structure of the carbon phase to become more orderly, leading to an increased degree of graphitization with decreased crystallite size [44].

### 3.2. Density

Table 2 depicts the densities of W_2_B_5_/C composites with different SiC contents, while the W_2_B_5_ content remains constant at 15%vol. The results show that as SiC content increased, the densities of composites also increased. Ultimately, the densities of the W15S30 composite reached 99.2%.

The introduction of Si powder in the composite system enhanced sintering by promoting the liquid phase, improving fluidity between particles, accelerating migration and rearrangement, and densifying the composites. The density gradually increased with a higher proportion of Si powder, peaking at 99.2% with a 30 vol% proportion. The densification improvement was mainly attributed to Si powder’s liquid-phase sintering during high-temperature hot-pressing, aiding particle rearrangement and plastic flow. External pressure played a role in enhancing contact between composite particles, leading to plastic yielding, increased reactant area, faster substance migration, and improved volume and grain boundary diffusion. This process helped reduce pores and internal defects in composites. During sintering, SiC and W_2_B_5_ particles adhered to the carbon-phase matrix surface, aligning with carbon-phase deflection and embedding between layers. Continuous hot-pressing pressure induced plastic flow deformation in the carbon-phase matrix, decreasing large pores to small or eliminating them entirely. Moreover, SiC and W_2_B_5_ undergo diffusion rearrangement during sintering, effectively filling residual pores within the matrix.

As the Si powder content in the reaction system increased, the volume effect resulting from the reaction of C and Si to form SiC also increased. In the sintering system, there was a significant presence of free C and free B. Liquid Si formed a solid solution with B and C, enhancing the volume diffusion of powder particles and distorting the B_4_C crystal structure. Due to the close proximity of Si to B and C in the periodic table and their similar properties, Si readily formed a solid solution with B_4_C, lowering the grain boundary energy of B_4_C and hastening its decomposition [45]. Additionally, Si segregated on the B_4_C grain boundary, increasing the surface energy of the ceramic particles and facilitating sintering densification. Consequently, with increasing SiC content, the material density experienced a notable enhancement.

As for calcined petroleum coke, it was challenging to sinter it into a dense block. In this experiment, the ceramic phase of W_2_B_5_ and SiC facilitated the sintering of the C phase. The reasons were as follows: (1) The reactions between B_4_C and WC and Si and C were exothermic reactions, and as the reactions proceeded, the thermal insulation temperature of the composite sintering system was enhanced, thereby promoting the sintering of the C phase. (2) Additionally, the presence of B and Si atoms in the composites positively influenced the graphitization of the carbon phases. These atoms enhanced the inter-layer positioning of carbon during the sintering process, facilitating the transformation of C-phase graphite and increasing the degree of graphitization. (3) Furthermore, W_2_B_5_ and SiC adhered to the C-phase particles; then, under external forces, they increased the contact among carbon phases, which facilitated plastic yielding. Various creep mechanisms contributed to migration, including volume diffusion and crystal lattice diffusion, which accelerated densification. (4) Lastly, carbon particles were situated between W_2_B_5_ grains, inhibiting the growth of these grains and playing a critical role in promoting density and enhancing strength.

### 3.3. Microstructure

**Figure 3:**
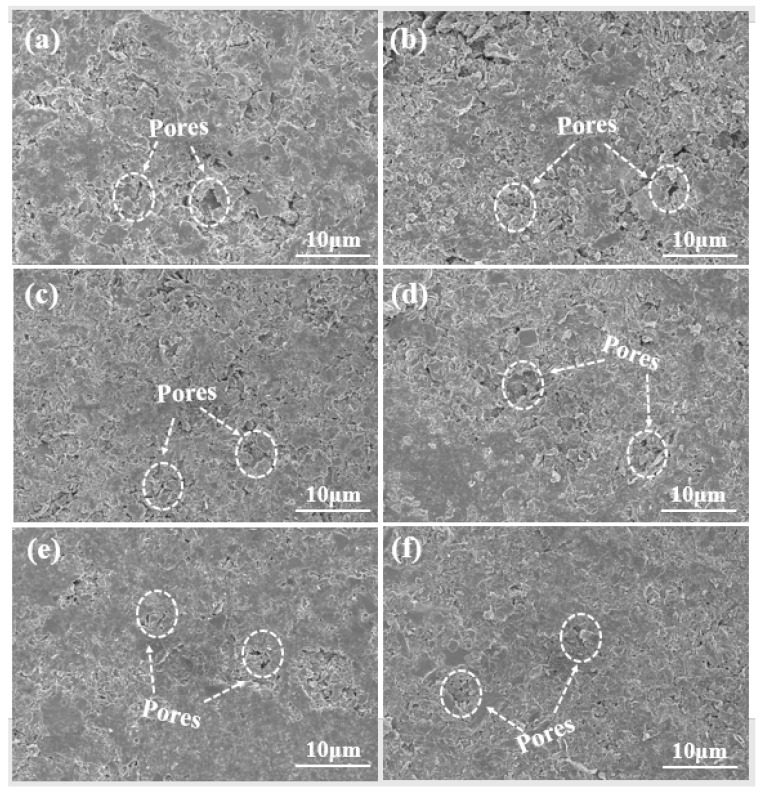
Surface morphologies of SiC-W_2_B_5_/C composites: (**a**) W15S5, (**b**) W15S10, (**c**) W15S15, (**d**) W15S20, (**e**) W15S25, and (**f**) W15S30.

Figure 3 illustrates SEM images of SiC-W_2_B_5_/C composites, showcasing a smooth surface, dense structure, and limited pores. The carbon-phase lamellae were observed to be flat, overlapping, and arranged in layers. Furthermore, the presence of SiC and W_2_B_5_ particles within the carbon-phase lamellae resulted in an interlocking structure that filled gaps in the graphite matrix.

**Figure 4:**
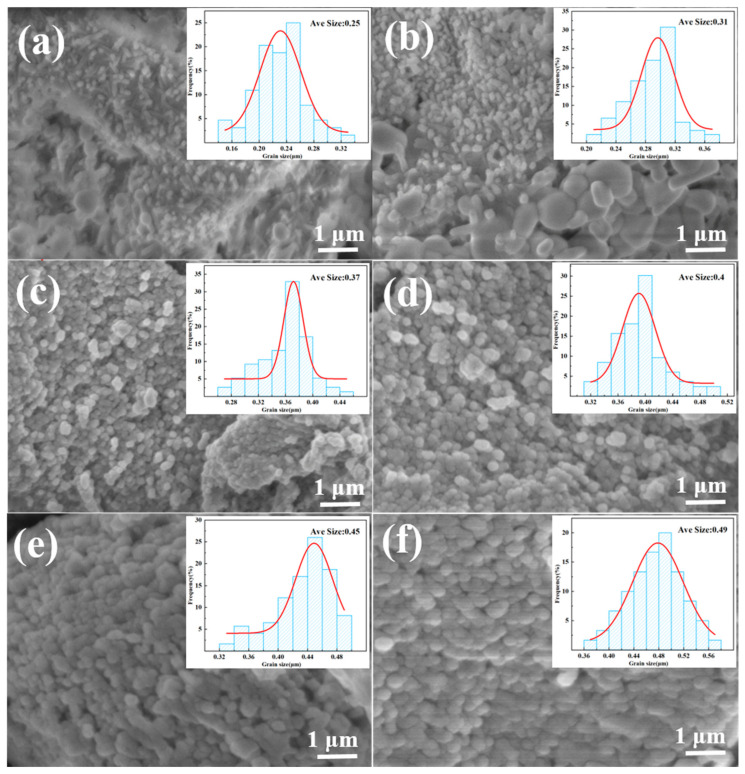
Crystallite size of SiC-W_2_B_5_/C composites: (**a**) W15S5, (**b**) W15S10, (**c**) W15S15, (**d**) W15S20, (**e**) W15S25, and (**f**) W15S30.

As the Si content increased, the porosity of composites gradually decreased. The interaction of Si and W_2_B_5_ during the pressing process caused slow flow in the carbon phase, resulting in a reduction in pore size, a denser microstructure, and a significant improvement in the mechanical strength of composites. In Figure 3a, the W15S5 composite is shown to have few pores due to insufficient sintering density, while the W15S15 composite showed a decrease in both the number and size of pores, as seen in Figure 3c. The W15S30 composite was the densest among these. With increasing Si content, the microstructure of composites became more uniform, as depicted in Figure 3g. The Si liquid-phase sintering process induced the flow of matrix particles, leading to smaller pores, a denser structure, and a substantial increase due to the liquid-phase effect of Si, which promoted the sintering growth of W_2_B_5_. The volume effect resulting from the reaction of carbon and Si drove W_2_B_5_ to migrate closer in liquid Si, reducing the distance between particles and enhancing bridging and growth [46]. Consequently, the final structure exhibited a trend of increasing W_2_B_5_ grain size. Figure 4a indicates that at 5 vol% Si content, the W_2_B_5_ particle size was approximately 0.25 μm, suggesting that lower Si content led to smaller W_2_B_5_ particle sizes during sintering. With Si content increasing to 15 vol%, as seen in Figure 4c, the volume and thermal effects from the reaction with C to form SiC intensify, reinforcing bridging between SiC and W_2_B_5_ particles. This transition from point contact to surface contact resulted in the formation of new interfaces between particles, leading to grain growth of 0.49 μm, as seen in Figure 4f. During the sintering process, Si melted into a liquid phase and B_4_C, and WC dissolved in the liquid alloy, exhibiting high activity. In the critical nuclear concentration zone, W_2_B_5_ was generated with W and B atoms, accompanied by the dissolution of WC and B_4_C. The behavior of B atoms was not constrained by gravitational forces. Consequently, during periods of strong convection and significant mass transfer, energy transformations occurred within the internal energy of the system. When the liquid phase was present, the concentrations of B and W reached elevated levels. Under conditions of excessive cooling and the coldness of the components, W_2_B_5_ crystal nuclei will form. The increase in diffusion coefficients further promoted the growth of W_2_B_5_ grains.

### 3.4. Strength and Toughness

By performing mechanical property tests on SiC-W_2_B_5_/C composite materials, this study established a correlation between organizational structure and mechanical properties. This paper also summarized the strengthening and toughening mechanisms of composites. The flexural strength, fracture toughness, and hardness of SiC-W_2_B_5_/C composites were individually assessed and the results are presented in Table 3.

It was evident that the W15S5 composite exhibited the lowest flexural strength due to the presence of micropores weakening the matrix, leading to easy breakage along the edges with a low strength of 154.5 MPa. As the Si content increased, the flexural strength of the SiC-W_2_B_5_/C composites also increased, with W15S30 showing a significant improvement to 292.3 MPa. The addition of SiC notably enhanced the material’s strength by reinforcing the matrix and reducing porosity. SiC, known for its high flexural strength, reinforced the matrix, further enhancing the composites’ strength [47]. The incorporation of SiC filled the material matrix pores, reducing porosity, increasing the effective stress-bearing area, and ultimately improving the flexural strength of the composites. The flexural strength was closely tied to their porosity, as stress concentration occurred at pores, leading to crack formation and propagation when stress surpassed a critical value. Minimizing pores increased the effective cross-sectional area under external force, enhancing the material’s stress-bearing capacity and strength.

The mechanical properties of the composites were also influenced by their composition. The structure and performance of composites were greatly affected by SiC content, which changed the volume fractions of ceramic and carbon phases, ultimately impacting their flexural strength. The strong bonding force between SiC and carbon was achieved through reaction sintering, involving the melting, diffusion, and reaction of Si at high temperatures with the carbon phase.

As the SiC content increased, the fracture toughness of composites also increased. For instance, at 5 vol% SiC content, the fracture toughness measured 2.62 MPa·m^1/2^. This value significantly rose to 6.12 MPa·m^1/2^ with a SiC content of 30 vol%. The addition of SiC had a positive effect on improving the toughness of composites. The fracture toughness increased with higher SiC content, as well-combined SiC formed through an in situ reaction with graphite matrix contributed to a significant toughening effect for W_2_B_5_/C composites.

The data showed a notable increase in hardness as the SiC content increased. For instance, the hardness of the W15S5 composite was 1.62 GPa, whereas the W15S30 composite exhibited a much higher hardness of 3.32 GPa. Porosity, grain size, and phase composition were the primary factors influencing the material’s hardness. The introduction of W_2_B_5_ and SiC through in situ reaction ensured uniform dispersion, aiding in sintering densification. The sintering process facilitated a reaction between Si and C, removing C to enhance hardness and yielding high-hardness SiC. The inclusion of SiC enhanced the material’s density, reduced porosity, and ultimately boosted hardness [48]. Moreover, both W_2_B_5_ and SiC serve as high-hardness materials themselves, further enhancing the hardness of composites.

### 3.5. Fracture and Morphology

**Figure 5:**
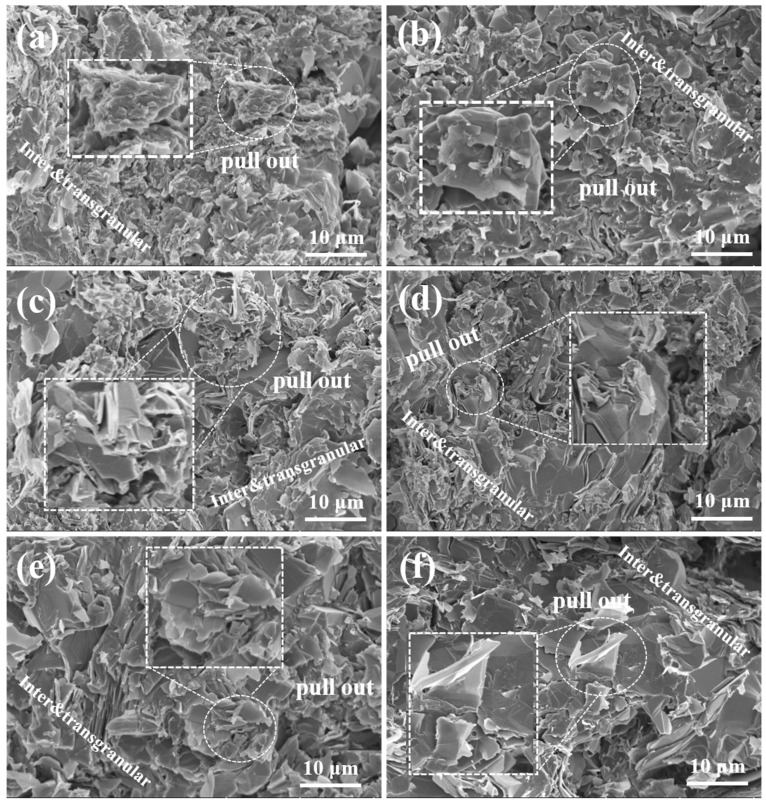
Fracture surface morphology of SiC-W_2_B_5_/C composites: (**a**) W15S5, (**b**) W15S10, (**c**) W15S15, (**d**) W15S20, (**e**) W15S25, and (**f**) W15S30.

Figure 5 illustrates the fracture morphology of SiC-W_2_B_5_/C composites. The fracture section showed a layered structure of the carbon phase, with SiC and W_2_B_5_ particles dispersed between graphite layers, enhancing reinforcement. The particles were closely packed without noticeable gaps, indicating good chemical compatibility between SiC and the carbon phase. During sintering, the SiC and W_2_B_5_ particles were evenly distributed on the surface of the C phase, working together to enhance bonding strength. SiC effectively prevented inter-particle fracture under external forces. Cross-sectional analysis provided insights into the microstructure and toughening mechanism of composites. As seen in Figure 5a, with SiC containing 5 vol%, low SiC content resulted in visible pores between particles, weak particle bonding, presence of coarse particles, incomplete elimination of pores in sintered body, and easy formation of pores at the carbon interface. This led to intergranular fracture and reduced strength of composites.

As the Si content increased, there was a gradual formation of SiC particles through a reaction, resulting in a decrease in material porosity. The composites in Figure 5b appeared denser, with sintered sample particles being thin, uniform, and showing low porosity and minimal defects. The cross-section looked relatively smooth, suggesting enhanced interface bonding strength between particles due to the presence of SiC. Various fracture modes existed in composites due to the interaction between the matrix and toughening phase [49]. The energy consumed during crack propagation by crack deflection caused through ceramic-phase particles, such as SiC and W_2_B_5_, led to strength enhancement. The combination of transgranular and intergranular fractures characterized the fracture mode of composites. Under external force, sliding friction at interfaces was higher than at weak grain boundaries. In the case of the W15S10 composite fracture, the pull-out of a peel-like dissociation surface indicated improved fracture toughness, attributed to intragranular fracture consuming more surface area than intergranular fracture. Transgranular fractures, mainly in large grains, were visible in Figure 5e,f, with pores in large grains leading to a higher proportion of transgranular fractures. Notable pores in the cross-section highlight the significant toughening effect of SiC-W_2_B_5_/C composites.

The SEM images illustrated the fracture surface of a composite with 30 vol% SiC. Figure 5f displays a seamless integration of SiC and carbon phases, with no clearly defined boundary visible. Both rough and smooth areas were present on the fracture surface, revealing uneven layers and pulled out particles. During the cleavage process, SiC particles were distinctly visible, suggesting a stronger interface compared to particle strength. The presence of SiC contributed to the growth of W_2_B_5_ grains, resulting in larger particles. These larger W_2_B_5_ particles experience transgranular fracture. The robust bonding between W_2_B_5_ grains and SiC led to crack propagation through ceramic-phase grains, resulting in transgranular fracture. Cracks rapidly propagate through the carbon phase due to weak binding. However, when cracks encounter ceramic-phase crystals, they circumvent the grains, elongating the crack propagation path and absorbing significant energy. This combination of transgranular and intergranular fracture modes enhanced the material’s toughness.

**Figure 6:**
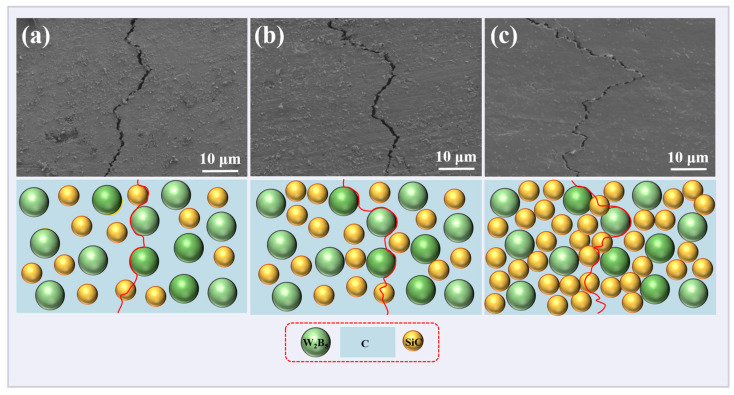
Crack propagation path of SiC-W_2_B_5_/C composites: (**a**) W15S5, (**b**) W15S15, and (**c**) W15S30.

The crack propagation paths of SiC-W_2_B_5_/C composites are depicted in Figure 6. The presence of plate-like W_2_B_5_ particles and SiC influenced fracture toughness, with higher SiC content resulting in increased crack deflection and a toughening effect from the drawing and bridging of plate-like grains. This led to a change in the crack propagation path, with increased energy consumption reducing crack propagation along crystals. As in Figure 6b, the shift toward the interface of the second-phase particles altered the fracture process to a combination of intergranular and transgranular processes, enhancing the toughness of composites. Higher SiC content increased the probability of contact with reinforced particles during crack propagation, leading to increased deflection and improved fracture toughness [50]. Furthermore, in Figure 6c, higher SiC content promoted a more evident grain growth trend, where plate-shaped grains pinned grain boundaries, allowing for even grain growth with fewer defects, directly impacting crack occurrence, strength, and toughness.

**Figure 7:**
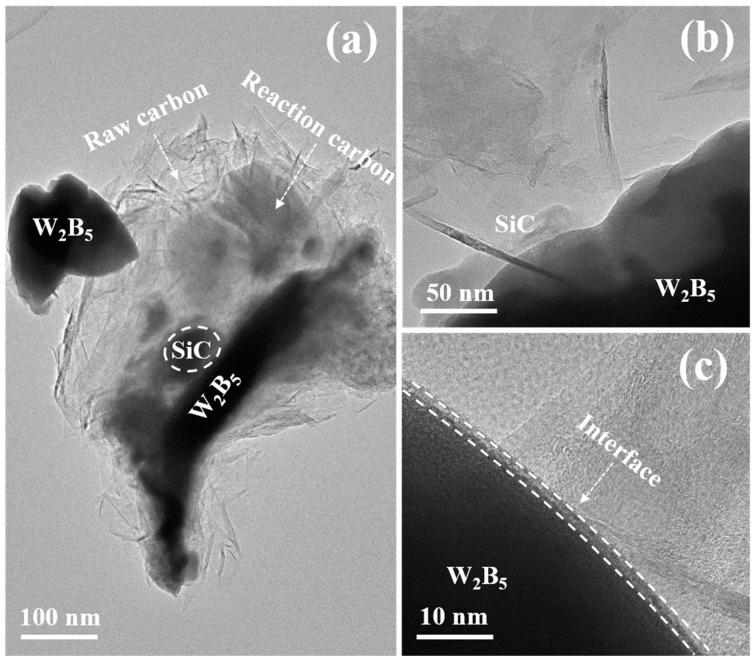
TEM images of the W15S15 composite. (**a**) TEM of W15S15, (**b**,**c**) HRTEM images of W15S15.

The surface of the carbon material was inherently inert, making it challenging to sinter. However, the Si powder in the raw material created a liquid phase at elevated temperatures, facilitating rearrangement and filling the gaps between carbon material layers. Through prolonged exposure to high temperatures and pressures, Si and C undergo an in situ reaction to produce SiC, thereby decreasing the pores within the matrix and promoting densification. The TEM morphology of the composite with 15 vol% Si powder content is illustrated in Figure 7. It depicts the distribution of phases in the samples of SiC (light-colored area), W_2_B_5_ (dark-colored area), and C (gray-white area). SiC particles were uniformly dispersed in the graphite phase. Upon melting, the Si powder diffused between the graphite sheets, reacting in situ to form SiC. The majority of the SiC maintained a quasi-flake structure, dispersed in the carbon phase matrix, filling the matrix’s voids and densifying the microstructure during hot-pressing.

During the sintering process, the temperature rise caused the Si powder to melt and transition into liquid Si. This liquid Si could efficiently infiltrate the C matrix, triggering a reaction that created a more refined structured reaction layer. As the reaction advanced, B_4_C gradually dissolved and reacted, culminating in the creation of a solid solution phase between B_4_C and Si within the C matrix and W_2_B_5_. The resulting phase composition was Si-B-C, as B_4_C dissolved in liquid Si and underwent a reaction. Figure 7 depicts the microscopic morphology of the reaction layer formed from the interaction between B_4_C and Si. The boundaries of the SiC region in the image appeared indistinct, making it difficult to differentiate between the adjacent SiC regions. The fully developed SiC structure showed a high density with minimal visible porosity. The rapid diffusion of C and B atoms in the Si liquid aided in the dissolution of the B_4_C matrix, resulting in an increased C concentration in the Si liquid. This higher C concentration promoted the nucleation and growth of SiC. The swift diffusion of C atoms within the liquid facilitated the movement of C from areas of high concentration to low concentration, supporting the nucleation and growth. Ultimately, a well-defined, dense, and elongated rod-shaped SiC structure formed within the reaction layer. The B_4_C matrix completely dissolved in the Si liquid, maintaining a constant total content of B and C. After diffusion, the distribution of B and C in the Si liquid became uniform, resulting in a reaction layer with a consistent structure. Further increase in content led to the continuous growth of SiC within the Si liquid through dissolution and precipitation. Small-sized SiC dissolved, while C atoms in the Si liquid nucleated and precipitated on the surface of large-sized SiC, eventually shaping them into long rods.

### 3.6. Graphitization

X-ray diffraction analysis was conducted to measure the (002) interplanar spacing of carbon in the SiC-W_2_B_5_/C composites; the degree of graphitization (*g*) followed the calculation in Formula (8).

(8) $$g=\frac{0.3440-d_{002}}{0.3440-0.3354}$$

where *g* (%) is the degree of graphitization of the sample, 0.3440 is the average spacing of ungraphitized portions, 0.3354 is the ideal graphitized layer spacing of complete ordered overlap, and *d_002_* is the face spacing of the sample [51].

As illustrated in Table 4, the results demonstrated that the incorporation of Si enhanced graphitization of the carbon phase within composites. This enhancement can be primarily attributed to the presence of a liquid phase formed by Si powder in the material’s sintering system, which facilitated the graphitization process of the carbon phase. The microcrystalline structure parameters of composites, as determined from XRD spectrum analysis and calculations, are presented in Table 4. At a Si mass fraction of 10% in the composite system, there was a notable increase in the degree of graphitization of the carbon phase. This suggested that Si doping had a promoting effect on the graphitization process of carbon under these conditions.

The SiC-W_2_B_5_/C composites prepared in this experiment were based on carbon (C). After these reactions, the volume content of the C phase accounted for 55–80 vol%. During the reaction process, Si consumed only a small portion of the carbon (taking the W15S5 as an example, the Si consumed only 1.4 g C during the reaction, while the added carbon source and reaction carbon totaled 52.6 g). Therefore, the matrix of composites was still dominated by C.

The carbon phase utilized in this study was calcined petroleum coke, which contained highly graphitized microcrystalline carbon as well as a significant number of cross-linked carbon bonds and interlayer defects. Graphitization transitions can occur following higher temperature treatment. In the case of the SiC-W_2_B_5_/C composites, the addition of W_2_B_5_ and SiC reduced the graphitization transformation temperature of the C phase. This addition facilitated the opening of cross-links within the internal structure and aided in eliminating defects, thereby accelerating the transition of disordered carbon structures to a three-dimensional ordered graphite structure. This process enhanced the graphitization of the C phase. Furthermore, with the incorporation of the W_2_B_5_ and SiC ceramic phase, B and Si atoms could infiltrate the graphite layers of carbon materials, replacing carbon to form a solid solution. This promoted changes in the graphite structure, making the covalent cross-links in the microcrystalline chaotic structure more susceptible to breaking, thereby catalyzing the reorganization of the carbon microcrystalline structure and facilitating the graphitization of carbon materials. The content of the W_2_B_5_ and SiC ceramic phase increased, leading to an expanded graphitization effect.

The main reactions in this system involved dissolution and elution of SiC. As the sintering system’s temperature surpassed the melting point of Si, solid Si particles absorbed enough heat to undergo a phase transformation, forming liquid-phase Si. Upon contact with the solid carbon phase, the dissolution reaction of the carbon phase in liquid Si occurred first. This exothermic process can elevate the temperature in the dissolution area. The initial stage occurred at the interface between solid particles and liquid Si, where the dissolution mechanism governed the reaction process. The introduction of liquid Si resulted in the dissolution of solid particles, leading to the formation of solid-phase precipitation upon reaching saturation. Both the dissolution of carbon and precipitation of SiC were exothermic reactions, releasing a significant amount of heat that increased the system’s temperature and carbon content. The solubility of carbon in liquid Si rose with temperature, aiding the solid solution of carbon. The precipitation of SiC from liquid Si was an endothermic process, establishing a temperature and concentration gradient within a reaction in the sintered body. Carbon diffused from regions of higher concentration to lower concentration, saturating in cooler areas and preferentially precipitating on the surface of solid carbon to create SiC grains. As temperature increased, carbon continued to dissolve, allowing for the even nucleation and growth of SiC in liquid Si. Once carbon was fully dissolved and saturated, SiC precipitated until supersaturation was removed. Carbon in liquid Si existed in various forms like C-Si groups and C-Si units. The precipitation units demonstrated strong adsorption at the interface between solid carbon and liquid Si, forming an adsorption layer mainly composed of SiC. This continuous adsorption layer at the interface enabled the constant diffusion of carbon through the SiC layer into liquid Si, leading to a continuous dissolution and release reaction.

The interaction between Si and B enhanced the catalytic effect on graphitization, leading to a higher degree of graphitization. The liquid phase formed was a carbon–graphite eutectic solution with carbon supersaturated in Si, SiC, W_2_B_5_, and graphite eutectic. This promoted the recrystallization of graphite, transforming the structure of carbon material from disorder to perfection. As the reaction progressed, the disordered carbon dissolved into supersaturated solution, allowing perfect graphite crystals to crystallize, catalyzing graphitization and reducing disorder in carbon material [52]. This conversion resulted in an increased degree of graphitization.

**Figure 8:**
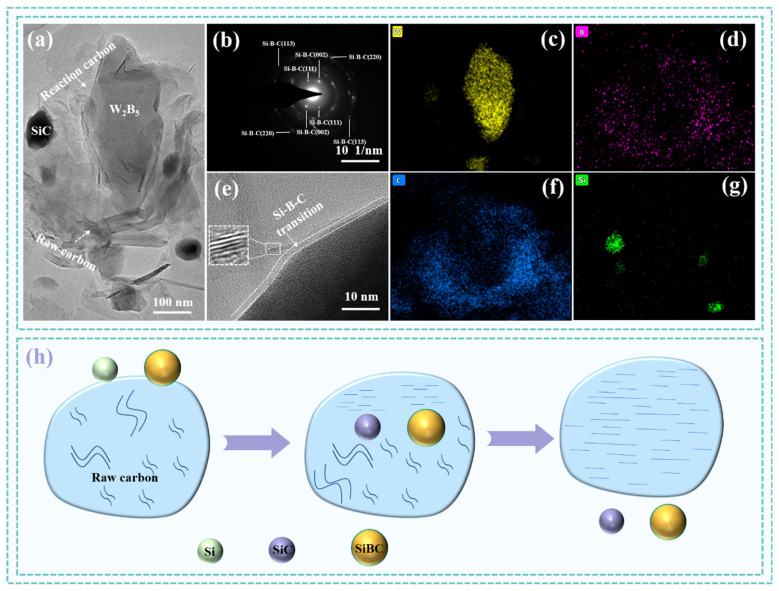
TEM images and graphitization mechanism schematic of the W15S15 composite. (**a**) TEM of W15S15, (**b**) SAED images of SiBC, (**c**,**d**,**f**,**g**) elemental maps of W, B, C and Si. (**e**) HRTEM images of W15S15, (**h**) graphitization mechanism schematic of SiC-W_2_B_5_/C composites.

Figure 8 presents HRTEM and SAED images, as well as the graphitization mechanisms of the W1515 composite. The atomic arrangement in the upper left corner of Figure 8e displays a disordered structure, indicating that it was an amorphous C phase, while the lower right corner reveals a ceramic phase. The C phase exhibits a band structure arranged at the interface. The graphitization transformation occurred, resulting in a transition layer at the ceramic interface. An analysis of Figure 8b showed that the crystal surface corresponded to the diffraction of Si-B-C in the [113], [111], [002], and [220] directions, with the diffraction of the crystal band shaft being evident. Furthermore, EDS analysis was conducted to determine the distribution of elements, as illustrated in Figure 8c–g, revealing numerous low-contrast particles within the continuous C phase matrix that confirmed these particles were W_2_B_5_- and SiC-phase particles. The element distribution indicated that the boundary between the W_2_B_5_ and the C phase was relatively indistinct. Additionally, an analysis of elements surrounding the C particles showed an enrichment of B and Si, suggesting the formation of the Si-B-C solid solution phase. The high-temperature sintering conditions facilitated the diffusion of Si and B into the C phase, thereby promoting the graphitization of the C phase. As demonstrated in Figure 8h, the process of Si-B-C-catalyzed graphite primarily involved the dissolution of the precipitation mechanism. The non-crystalline disorders present in the composite’s carbon phase can be dissolved within the Si-B-C. In this context, when the carbon became overly saturated, the dissolved portion of the carbon was referred to as carbonized graphite, which distinguished it from orderly and disorderly carbon. The Si-B-C atom played a crucial role in the carbon crystal lattice through diffusion. During the sintering process, although Si-B-C diffused similarly within the carbon crystal lattice, its diffusion rate was significantly higher than that of carbon. This enhanced diffusion rate facilitated the growth and development of graphite microcrystals, thereby increasing the graphitization. During the liquid-phase sintering process, B_4_C particles dissolved in molten Si. As the concentrations of B atoms and C atoms in molten Si saturate increased, a Si-B-C transition phase rich in Si and B was formed, as shown in Figure 8. The creation of a Si-B-C solid solution phase resulted from an interaction between B_4_C particles and Si during sintering. As Si melted during sintering, it formed liquid Si with a relatively low concentration in B_4_C grains. Through diffusion along the concentration gradient, liquid Si migrated from high to low concentration areas within the B_4_C grain. The development of a Si-B-C transition phase reaction can be segmented into stages: Si initially combined with C in the B_4_C matrix to produce SiC, transforming B_4_C into the B-rich-phase Si-B-C. Initially, the B and C content in the Si liquid was minimal. With increased penetration of Si liquid, the B-rich-phase Si-B-C dissolved in liquid Si, subsequently elevating the B and C content in the Si liquid. As the cooling process ensued and the system temperature decreased, saturated solubility of B and C in the Si liquid diminished. Alongside the existing SiC in the reaction layer, the B_4_C and C phases precipitated anew. Essentially, Si initially captured C from B_4_C to form SiC and Si-B-C, which then dissolved in the Si liquid and precipitated as SiC, B_4_C, and C. As the dissolution and precipitation reaction progressed, the level of graphitization in the C phase within the composite system gradually increased.

### 3.7. Oxidation Behavior

**Figure 9:**
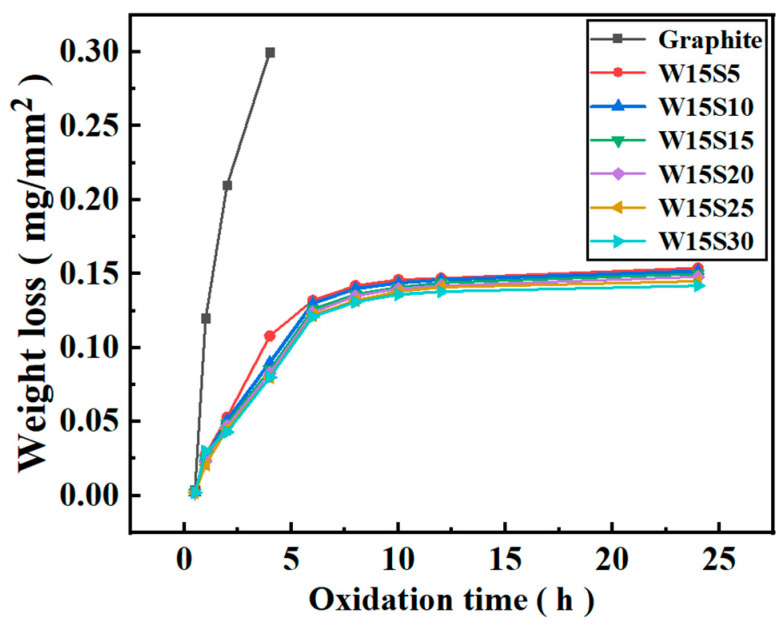
Calculated oxidation rate of W_2_B_5_/C composites.

Figure 9 illustrates the relationship curve between weight loss per unit area and oxidation time of SiC-W_2_B_5_/C composites after exposure to air at 600 °C for 24 h. The oxidation weight loss in the initial 6 h showed a nearly linear correlation with oxidation time, suggesting a gradual stabilization as oxidation progressed. This behavior was attributed to the preferential oxidation of carbon on the composite material’s surface during the early stages, due to its higher content compared to the ceramic phase. Consequently, W_2_B_5_ and SiC initially exhibited low oxidation levels, which hindered the formation of a protective oxide layer. As oxidation advanced, the oxidation degree of W_2_B_5_ and SiC increased, resulting in a consistent weight loss pattern. After 6 h, weight loss stabilized due to the formation of a protective film of B_2_O_3_ and SiO_2_ on the surface, which prevented further oxidation. The Si-B-O oxide layer, formed by the interaction of B_2_O_3_ and SiO_2_, effectively sealed the material’s surface, reducing pore formation and halting additional oxidation.

**Figure 10:**
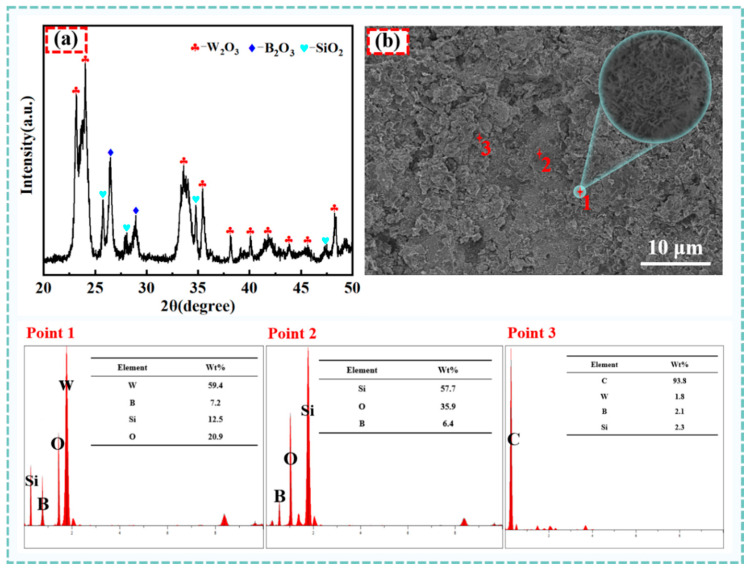
XRD patterns and EDS element analysis of the W15S15 composite oxidized after 8 h. (**a**) XRD patterns of W15S15 composite oxidized after 8 h, (**b**) EDS element analysis of W15S15 composite oxidized after 8 h.

Figure 10 depicts the XRD pattern of the W15S15 composite material surface after 6 h of oxidation. Figure 10a presents an XRD analysis of W15S15 at 800 °C. The oxidation surface revealed that the composite oxidized to form WO_3_, B_2_O_3_, and SiO_2_. As illustrated in Figure 10b, the oxidation surface appeared loose and porous, with the oxide layer composed of small, quasi-ball-shaped particles. EDS analysis indicated that these particles consist of the elements W, O, Si, and B. Combined with the XRD results, this supports the identification of the materials as WO_3_, SiO_2_, B_2_O_3_, and B_2_O_3_ in a liquid form, exhibiting local bonding that encapsulated WO_3_ and SiO_2_ particles. The white spherical particles corresponded to WO_3_, while the granular-like agglomerates represented SiO_2_. The lamellar material signified C, and the morphology of B_2_O_3_ was less distinct due to its liquid state, which bound to and encased the SiO_2_ and W_2_O_3_ particles. The continuous and dense oxidation layer effectively prevented O_2_ from penetrating the interior, thereby reducing the oxidation of each phase within the substrate and enhancing the composites’ antioxidant properties.

**Figure 11:**
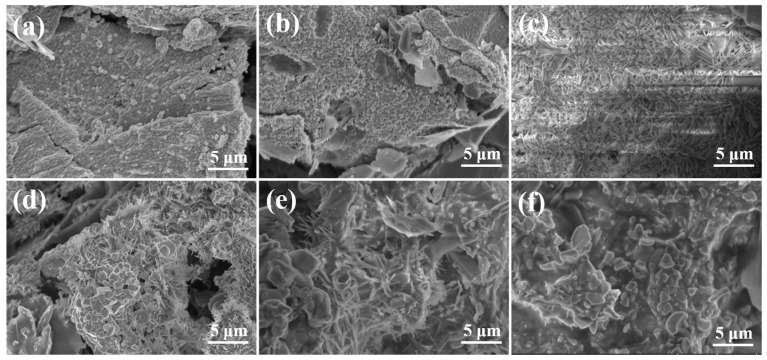
Surface morphology of the W15S15 composite after oxidation treatment: (**a**) 2 h, (**b**) 4 h, (**c**) 6 h, (**d**) 8 h, (**e**) 12 h, and (**f**) 24 h.

In the initial stages of oxidation, the loose and porous surface layer does not provide protection, as seen in Figure 11a. Oxygen can easily penetrate through these pores, react with carbon in the matrix, and increase weight loss. After 6 h of oxidation, a porous network structure consisting of interconnected glassy structures formed on the surface in Figure 11c. This was primarily attributed to the decreased viscosity of B_2_O_3_ and improved fluidity, leading to the B_2_O_3_ film covering the surface of the carbon matrix. Additionally, SiO_2_ dissolved in B_2_O_3_ to create a Si-B-O solid solution oxide layer film, effectively hindering oxygen diffusion into the interior and reducing matrix oxidation. As oxidation progressed, numerous white particles and spherical protrusions emerged on the oxidized surface, indicating the promotion of WO_3_ growth and the formation of a protective WO_3_ layer that hindered B_2_O_3_ volatilization and oxygen ingress, as seen in Figure 11d. This oxidation process of composite materials played a significant role in prevention. By the 24 h mark in Figure 11f, WO_3_ particles elongated into strips. The growth of WO_3_ grains demonstrated enhanced bulk diffusion and grain boundary diffusion at elevated temperatures, leading to rapid grain growth. The increased volume of WO_3_ further shielded the matrix from oxidation.

The oxidation process of SiC-W_2_B_5_ composites was influenced by the oxidation behavior of each component within composites. Initially, the surface carbon of the sample absorbed oxygen, leading to CO_2_ formation. This gas release enhanced the interaction between matrix carbon and oxygen, accelerating oxidation. Weight loss exhibited a linear correlation with oxidation time. During this phase, W_2_B_5_ underwent a slower oxidation rate, resulting in minimal generation of glassy B_2_O_3_. This insufficient filling of material defects, coupled with the low density of W_2_B_5_ and the voids left post-carbon oxidation, weakened its protective capabilities on the material. As the oxidation time increased, W_2_B_5_ began to oxidize, leading to the formation of oxidation products W_2_O_3_ and B_2_O_3_ that created a continuously distributed oxide layer on the composite material’s surface. This layer acted as a barrier, hindering the diffusion of oxygen into the material’s interior and consequently slowing down the oxidation rate. Subsequently, SiC also started to oxidize gradually, resulting in the generation of SiO_2_, which formed a thin-layer wrapping surface, and initiating the formation of a Si-B-O glass layer with B_2_O_3_. This glass layer not only inhibited the volatilization of B_2_O_3_ but also contributed to the creation of a protective oxide layer on the composite material’s surface, further impeding oxygen diffusion. Simultaneously, the rapid growth of WO_3_ particles led to the development of a dense WO_3_ layer and a Si-B-O glass layer, which together enhanced the material’s resistance to oxidation by preventing oxygen diffusion into the interior.

## 4. Conclusions

In this paper, SiC-W_2_B_5_/C composite materials were fabricated by adjusting the SiC proportion, resulting in a SiC content ranging from 5 vol% to 30% vol. The density of the composite materials increased with higher SiC content, reaching a maximum of 99.2% from an initial 96.1%. The W15S30 composite material exhibited a flexural strength of 292.3 MPa, fracture toughness of 6.12 MPa·m^1/2^, and Vickers hardness of 3.32 GPa. The SiC phase played a crucial role in toughening the composite material, resulting in a mixed fracture mode of transgranular and intergranular with a pull-out phenomenon. The interface between the ceramic and carbon phases showed good combination. During sintering, Si powder acted as a liquid-phase sintering agent, reacting with B_4_C to increase B concentration and promote W_2_B_5_ formation. Furthermore, Si powder reacted with carbon to form a transition phase of Si-B-C, enhancing interface bonding strength. Si powder also catalyzed the graphitization process of composites, with a more pronounced effect as SiC content increased, leading to an increase in the chemical degree of graphite from 59.2% to 66.2%, and the composites demonstrated excellent antioxidant properties, which was attributed to the formation of a protective layer on the surface.

## Figures and Tables

**Table 1:** Compositions of SiC-W_2_B_5_/C composites.

Sample	Raw Materials (wt%)				Reaction Products (Vol%)		
	WC	B_4_C	Si	P	W_2_B_5_	SiC	C
W15S5	40.8	7.2	3.3	48.7	15	5	80
W15S10	40.9	7.3	6.6	45.2	15	10	75
W15S15	41.1	7.4	9.7	41.8	15	15	70
W15S20	41.9	7.5	12.1	38.5	15	20	65
W15S25	40.6	7.3	17.5	34.6	15	25	60
W15S30	39.8	7.1	22.8	30.3	15	30	55

**Table 2:** SiC-W_2_B_5_/C composites.

Sample	Theoretical Density (g/cm^3^)	Density (g/cm^3^)	Relative Density (%)
W15S5	3.59	3.45	96.1
W15S10	3.64	3.54	97.3
W15S15	3.69	3.62	97.9
W15S20	3.77	3.71	98.3
W15S25	3.82	3.78	98.8
W15S30	3.86	3.83	99.2

**Table 3:** Properties of SiC-W_2_B_5_/C composites.

Sample	Flexural Strength (MPa)	Fracture Toughness (MPa·m^1/2^)	Vickers Hardness (GPa)
W15S5	154.5	2.62	1.62
W15S10	182.3	3.23	1.98
W15S15	211.5	3.92	2.32
W15S20	239.6	4.72	2.62
W15S25	265.4	5.43	2.95
W15S30	292.3	6.12	3.32

**Table 4:** Degree of graphitization of SiC-W_2_B_5_/C composites.

Sample	2θ (°)	D_002_/(nm)	Degree of Graphitization (%)
W15S5	26.64	0.33904	59.2
W15S10	26.69	0.33876	60.8
W15S15	26.78	0.33845	62.3
W15S20	26.83	0.33813	63.5
W15S25	26.88	0.33786	64.8
W15S30	26.92	0.33761	66.2

## Data Availability

The original contributions presented in this study are included in the article. Further inquiries can be directed to the corresponding author.
